# The Advent of Coronavirus Disease 2019 and the Impact of Mobile Learning on Student Learning Performance: The Mediating Role of Student Learning Behavior

**DOI:** 10.3389/fpsyg.2021.796298

**Published:** 2022-02-08

**Authors:** Zhiwei Wang, Alia Qadir, Alia Asmat, Muhammad Sheeraz Aslam Mian, Xiaoli Luo

**Affiliations:** ^1^Department of Economics and Management, Nanjing Agricultural University, Nanjing, China; ^2^College of Liberal Arts, Technological University of the Philippines, Manila, Philippines; ^3^Department of Management Sciences, Riphah International University, Faisalabad Campus, Faisalabad, Pakistan; ^4^Department of Psychology, University of Central Punjab, Lahore, Pakistan; ^5^Department of Business Administration, Allama Iqbal Open University, Islamabad, Pakistan; ^6^School of Economics, Central South University for Nationalities, Wuhan, China

**Keywords:** mobile learning, student learning behavior, student performance, collaborative learning behaviors, personalized learning behaviors, social learning behaviors

## Abstract

The recent coronavirus disease 2019 (COVID-19) pandemic pushed almost all institutions to adopt online and virtual education. The uncertainty of this situation produced various questions that perplexed educationists regarding what implications the pandemic would have on educational institutions, especially regarding how the switch to online education would impact the behavior and performance of students. The vast importance of this matter attracted the attention of researchers and served as the motivation for this research, which aims to resolve this confusion by studying the use of mobile learning (ML) among students for educational purposes during the COVID-19 period. This study also examines how this situation has affected student learning behavior (LB) and performance (SP) in the higher education setting. This research is based on collaborative learning theory, sociocultural learning theory, and ML theory. This quantitative research employed the convenient sampling technique to collect data through structured questionnaires distributed to 396 students of higher education institutions who carry a mobile device. This study used descriptive and inferential statistics to make the data more meaningful. Structural equation modeling (SEM) with AMOS software was used for hypothesis testing. The results showed that ML was a significant and positive predictor of SP and LB. Moreover, student LB partially mediated the relationship between ML and SP. The findings suggest that the academic performance of students can be enhanced by building a ML environment that aligns with the LB of students. Nevertheless, content suitable for ML must be developed, and future research should be conducted on this topic.

## Introduction

Many countries decided to close their educational institutions to reduce the spread of coronavirus disease 2019 (COVID-19) (Naciri et al., 2020). Although such closures were inevitable (Czerniewicz, 2020), they have threatened the future of students (Usak et al., 2020), as “this disruption of education could leave the children at risk of child labor, early marriage, exploitation, and recruitment into armed forces” (Baytiyeh, 2018). However, fortunately, due to the Internet, the world was already equipped with digital textbooks, digital libraries, interactive games, social media, electronic devices, and robotics that can be used for learning outside of the classroom. Mobile devices with Internet connectivity, such as smartphones, personal digital assistants, iPads, and tablets, have already drastically transformed many aspects of life and revolutionized the education industry. Therefore, the only solution seemed to be a complete shift from classroom learning to mobile learning (ML) at all academic levels. As such, this innovative learning methodology (Liguori and Winkler, 2020) became very popular and vital (Toquero, 2020; Alghazi et al., 2021), as it could help the students to decrease their study gap by serving as a substitute learning methodology during COVID-19 (Naciri et al., 2020). This change also opened several new avenues for teaching.

According to the literature, ML has been accepted as an efficient teaching and learning system (Kabir and Kadage, 2017), most notably because it enables the education process to be carried out at anytime from anywhere (Bidin and Ziden, 2013) with anyone. Students can access up-to-date materials *via* ML, which promotes collaboration and strengthens their engagement. Also, since mobile devices are easy to carry, it is easy for their owners to stay connected and obtain a broad range of educational and information sources at all times (Ismail et al., 2016).

Moreover, some evidence shows that school closures can produce significant losses in educational achievement, particularly for disadvantaged students (Eyles et al., 2020). Such evidence evoked fear that ML may not be as effective as face-to-face education. The purpose of this study was to resolve the contradiction regarding the workability or ineffectiveness of ML by investigating its effects on student learning behavior (LB) and student performance (SP) in higher education settings at a time when many governments have already announced emergency policies and suspended face-to-face classes due to the closures of educational center while promoting ML (Thomas and Cathy, 2020).

The researchers believe that if ML can change and improve LB, it will surely add to SP (Means et al., 2000). In the United States, the Department of Education conducted a meta-analysis comparing virtual learning with face-to-face learning. The analysis clearly indicated that the mean effect was a 24% higher SD for ML. This study also examines the fact that ML is not merely an alternative for use in the emergency situation caused by COVID-19. It may also be an effective means of delivering education under normal conditions, as it can augment face-to-face learning. Nevertheless, there is growing concern that ML will cause SP to decrease.

It has also been observed that ML can change the LB of students. If such observations are accurate, then ML can be implemented to enhance or reduce SP. However, to the best of the knowledge of researchers, no study has examined the relationships of ML with LB, and SP has not yet been checked. Thus, there is an urgent need to study these relationships to remove the confusion surrounding ML, which seems to have become a standard part of learning.

## Theoretical Background

Many theories of learning have been developed over the 2,500 years between Confucius’ time and the present day; almost all of them have been predicated on the assumption that learning occurs in a school classroom and is mediated by a trained teacher. This research is based on three theories. The first is *ML theory*; at present, ML involves much more than accessing learning content on mobile devices. Also, learners move from place to place, build or join groups and communities, and use robust personal technologies. Mobile devices and M-technologies ease interactions within an innovative context. Thus, ML makes use of shared technologies (Westera, 2011).

The second theory central to this study is Vygotsky’s (1978) *sociocultural theory* of human learning, which describes learning as a social process and that the origin of human intelligence is society or culture. The central theme of sociocultural is that social interactions play a fundamental role in the development of cognition. It further expounds that learning primarily occurs through interpersonal interactions and intrapersonal communication. That is, learners influence other learners *via* social interactions, and new ideas are generated by group and community discussions and by accepting the arguments of others.

The third theory that this research considers is *collaborative learning theory*. This theory says that collaborative learning is subject to the vigorous involvement, contribution, and reflective and insightful engagement of learners in ML, group contexts, group members, peer-to-peer communication, knowledge sharing, and help solving problems within a group. It accelerates interactions and facilitates participation among learners (Sarrab et al., 2013). In a collaborative learning setting, learners can converse with their peers, present and defend ideas, exchange diverse beliefs, and question other conceptual frameworks, all of which foster active engagement (Srinivas, 2011).

## Hypotheses Development

### Mobile Learning and Student Performance

Hashim et al. (2011) defined SP as the degree to which students are confident that M-technology usage is beneficial and suitable for education purposes by making it convenient for students to learn in diverse locations (Alqahtani and Mohammad, 2015). SP can be appraised by observing their activities and academic actions (e.g., whether their level of learning will be of higher quality, their subject understanding has expanded, planned learning outcomes are accomplished, learning through m-Apps is enjoyable, peer collaboration is enhanced, productivity is improved, and positive attitudes are displayed) (MacCallum and Jeffrey, 2009).

Mobile learning is perceived and defined differently by different people but most include terms such as “mobile technology,” “ubiquitous,” “mobility,” “individualism,” and “e-learning” (Keskin and Metcalf, 2011). ML can be defined as the learning or sharing of information *via* mobile devices that do not require the presence of learners at a predetermined location. A literature review shows that research on distance education (including online education) has been conducted in South Asian countries. Although a few indirect studies on ML have been carried out, it remains unclear how ML enhances LB and SP, especially in the COVID-19 period, during which almost all students have shifted to ML. ML has changed the lifestyles, thinking habits, learning abilities, and LB of learners. ML also provides significant advantages within direct and indirect education modalities (Hao et al., 2017). Mobile devices have refuted the concept that learning can take place only in the classroom by providing teachers and students with learning materials regardless of time and location (Jaradat, 2014). It seems that, in this way, ML will lead to better SP, hence the first hypothesis of this study:

**H_1_:** ML has a positive effect on the learning performance of students.

Furthermore, a dimensional study of the effect of ML on SP was needed because this research aimed to provide in-depth knowledge of this relationship. ML has three dimensions: learning mobility (LMO), learning self-efficacy (LSE), and learning motivation (LMT). ML influences the motivation, collaboration, information sharing, interactivity, and mobility of learners (Khaddage et al., 2009). Directly connected devices undoubtedly change LB and SP. ML allows students to instantly respond to questions posed by their fellow learners, friends, relatives, and instructors by utilizing their time efficiently to improve performance (Shorfuzzaman and Alhussein, 2016). Moreover, students can interact, communicate, and explore new information, which immensely increases their cognitive ability. As technology becomes more ubiquitous, the true effect of ML on mobility will continue to be observed and experienced.

**H_1_a:** Learning mobility has a positive effect on the learning performance of students.

Bandura (1997) described academic LSE as the belief of individuals that they can accomplish what is required of them per the educational task or achieve a clear educational goal. Li et al. (2018a) stated that LSE comprises confidence, technology experiences, the difficulty of using technology, and sources of confidence. The LSE of an individual is related to their smartphone usage and habits, computer literacy, Internet literacy, expertise, and self-confidence (Razzaq et al., 2018). Pajares (2002) argued that LSE in higher education “influences the choices students make and the course of action they pursue.” Therefore, it is a pivotal contributor to SP (Tsai, 2012). ML self-efficacy provides a way for learners to increase their participation, engagement, and self-confidence; hence, it has a positive effect on SP (Yukseloglua and Karaguven, 2013).

**H_1_b:** LSE has a positive effect on the learning performance of students.

Motivation is essential for engaging learners, promoting learning effectiveness (Malone and Lepper, 1987), and involving and sustaining mobile device use for ML. Motivation fosters interaction and excellent performance among students [collaborative LB (CLB)]. Motivation is based on the changes in the learning style of the student. The student can seek, gather, and share information at any location at any time with anyone [social LB (SLB)]. Motivation fosters collaboration and interaction among learners (Kilis, 2013), and highly motivated learners can effortlessly organize their learning tasks when using ML. Strong motivation is essential to comprehend a subject (*via* rehearsal and self-regulation), excellent learning, and attain the best performance (Li et al., 2018b).

**H_1_c:** Learning motivation has a positive effect on the learning performance of students.

### Mobile Learning and Student Learning Behavior

Mobile learning affects learners through information seeking, information gathering, information sharing, coordination, and collaboration. The availability of information and learning has become much broader due to technological developments. Teachers and learners have become more professional, better critical thinkers and problem-solvers, and more creative and knowledgeable. In addition, the attitudes and behaviors of students are changing from their use of mobile technology. Specifically, literacy, numerical skills, and recognition of their abilities of learners have improved. Such changes have increased the self-esteem and self-confidence of learners and encouraged independent learning.

Learning anything at anytime from anywhere reflects the autonomy of learners (i.e., student-centered personalized learning) (Traxler, 2005), person-to-person (peer-to-peer, collaborative), cooperative, and communicative interactions, motivation to learn (LMT), immediacy and flexibility, convenience, accessibility, and cost-effectiveness (Valk et al., 2010). ML has transformed this generation of students into digitally literate learners who are socially connected to others in immediate and experimental learning environments. ML has significantly impacted the achievements, self-regulation [personalized LB (PLB)] (Zare Bidaki et al., 2013), and conversational skills of students (Elfeky and Masadeh, 2016). It has also led to significantly increased interactivity in large blended classes while helping learners become more behaviorally, intellectually, and emotionally engaged in learning activities (Wang et al., 2009). According to the above discussion of the extant literature, the following hypothesis is proposed:

**H_2_:** ML has a positive effect on the LB of students.

### Student Learning Behavior and Student Performance

Learners engage in various activities and perform various LBs in ML settings, and the use of ML (apps) affects LB (Alqahtani and Mohammad, 2015) through the acquisition of skills and knowledge on mobile devices at any time, from anywhere (Behera, 2013). LBs are the activities associated with the process of learning, for example, communication (SLB), interaction (CLB), self-motivation (PLB), and satisfaction with education (Dillon et al., 2007). LBs associated with ML are habitually used in social network-based learning (Koole, 2014) (SLB) and in virtual learning communities in cooperative-collaborative online settings (Yang et al., 2015). Thus, ML enhances the propensity of the learner to become involved in personalized self-directed, self-regulated learning. ML aids the growth of the vital 21st-century skills of learners, including collaboration and communication, critical thinking and problem-solving, digital literacy, and creativity.

**H_3_:** Student LB has a positive effect on SP.

Mobile learners achieve social learning through social networks and online communities, engage in peer-to-peer collaborative learning, find answers from the Internet, submit assignments through email, and organize their work using technology, thus performing personalized learning tasks. Learners can share information and collaborate with their peers. LB is comprised of PLB, CLB, and SLB. In PLB, students manage their learning *via* self-regulation while monitoring their thoughts and acting to achieve set objectives. However, gaining a thorough understanding of this relationship is one of the objectives of this research; therefore, a dimensional study of LB on SP was carried out.

Personalized LB manifests in self-direction, self-empowerment, and preparedness for learning. Meanwhile, cocreating tailored learning is customized to the needs, interests, and strengths of learners while the learners regulate what, how, when, where, and with whom they will learn. The learners also create the content and complete assignments with their peers (Nilsson, 2016). While the self-control of learners is theoretically appealing, its impact is consistent in empirical studies (Admiraal et al., 2018).

**H_3_a:** Personalized LB has a positive effect on SP.

The second dimension, i.e., collaborative learning, is when students intentionally work to accomplish common goals (Pellas et al., 2013). It is “a situation in which two or more people learn or attempt to learn something together” (Cruz-Flores and López-Morteo, 2008). The learning performance of students improves from social interactions with their fellow students (Kreijns et al., 2003). M-technologies enhance the independence and courage of learners that they can collaborate with others when learning (Wang and Wu, 2008; Al-Rahmi et al., 2015). M-technologies also support the notion that cooperative learning is an active pedagogy that fosters academic achievement. CLB involves intricate descriptions, getting and giving assistance, negotiating, accepting contrasting viewpoints, helping mobile learners act productively in groups, and affecting the academic performance of learners. Collaborative learning results in higher levels of achievement and productivity. CLB provides learners with additional responsibility and control over their learning outcomes in addition to encouraging teamwork, improving interpersonal competence, and enhancing performance (del Barco et al., 2017).

**H_3_b:** Collaborative LB has a positive effect on SP.

Social LB can be referred to as an alteration in knowing beyond one person that occurs inside broad social groups, social units, or communities *via* social interactions between individuals in social networks (Reed et al., 2010). Schwen and Hara (2003) stated that social learning takes place when students with joint interests form groups, share ideas, and find solutions. Social learning is crucial at several levels, predominantly when a network helps a learner to create bonds with other learners within broad social communities. Belonging to a community of fellow learners and peers (network of support) motivates learners to attend the lessons and class contribution in the community system.

Concerning the relationship between SLB and SP, studies over recent decades show that social competence and academic achievement are interrelated, not discrete (Wentzel, 1991a,b, 1993; Caprara et al., 2000; Welsh et al., 2001). Similarly, the number of researches examining the impact of social learning on students (performance) has increased. Zins et al. (2004) concluded that social learning affects the summative achievement and performance of students. Hence, this study aimed to determine how learners improve their performance by applying SLB within an ML perspective. Hence, we posit the following hypothesis:

**H_3_c:** Social LB has a positive effect on SP.

### Meditating Role of Student Learning Behavior

Social LB can be referred to as an alteration in knowing beyond one person and occurs inside widespread social groups, social units, or communities through social interactions within social networks (Reed et al., 2010). Communities of fellow learners and peers (network of support) motivate attendance for lessons and class contributions. According to the self-regulated learning theory, learning strategies and motivation directly influence learning outcomes. If the improvement in LB is attributed to ML, then LB will have affect SP. In this case, it can be said that academic involvement is achieved through the social affiliations among learners (Tinto, 1994), thus leading to the following hypothesis:

**H_4_:** Student LB mediates the relationship between ML and SP.

**FIGURE 1:**
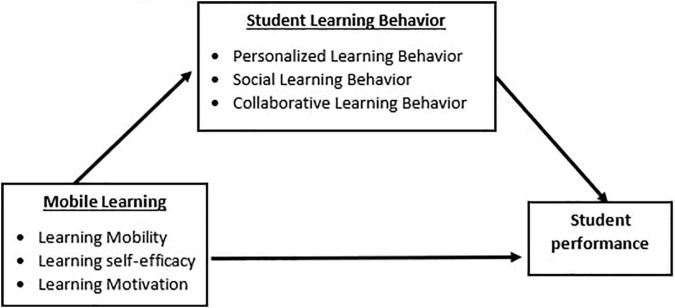
Conceptual framework.

The conceptual framework is given in Figure 1.

## Materials and Methods

This quantitative research was carried out in the educational sector, more specifically, at higher education institutions in Faisalabad and Islamabad. Its main objective was to study the effects of online education on the behavior and performance of students among university students who took online classes during the COVID-19 lockdown. These students were selected because almost all university students own either a mobile device or laptop which they use for their independent educational use. Most college students in developing countries do not own their mobile devices and depend on their parents (especially their fathers) to supply these devices. Therefore, they can use mobile devices for educational purposes only when their fathers are at home with them. Per the objectives of this research, it can be conducted only on university students who were free to use mobile devices for their education at their convenience.

This cross-sectional study employed a predictive non-experimental survey design. Data were collected through self-administered questionnaires, with items rated on a 7-point Likert scale. The painstaking efforts were made to collect data from students attending different higher educational institutions to ensure that the sample is truly representative of the study population. Hatcher (1994) suggests that the number of participants should be approximately 10 times the number of measurements used in an structural equation modeling (SEM) analysis. Since the survey used in this study contained 41 items, the suitable sample size is 410. A convenience sampling technique was used to determine the sample. After the preliminary screening of the survey questionnaires, and following Malhotra (2010), 396 out of 430 questionnaires were selected for the analysis. Thirty-four questionnaires were discarded because they seemed to be carelessly, incorrectly, or incompletely filled out. The data were first entered in the SPSS data sheet and then tested for multivariate assumptions.

### Sample Profile

The sample of 396 students comprised 51% (202) men and 49% (194) women. Also, 64.9% (257) were full-time students, and 35.1% (139) were part-time students. Of the participants, 64.89% (257) used mobile devices for their online studies, whereas 35.10% (139) used laptops for this purpose. Regarding age, 63.3% (250) aged 18–22 years, 31.8% (126) aged 23–27 years, and 5.05% (20) aged 28 years and above.

### Measurements

The survey questionnaire consisted of 45 items, comprising four demographic items, namely, gender, age, studentship category, and mobile device ownership, as all of these were the important antecedents of ML (Qureshi et al., 2002). The rest of the variables were measured *via* adopted scales with confirmed reliability and validity. The LB construct was measured using an adapted Motivated Strategies for Learning Questionnaire (MSLQ) by Kukulska-Hulme and Traxler (2007), which has three dimensions. The first dimension is CLB (six items); a sample item is “I was able to develop problem-solving skills through peer collaboration.” The second dimension is SLB (six items); a sample item is “My learning by using Social Media (WhatsApp, Facebook, Twitter, YouTube, …) makes learning easy.” The third dimension is PLB (16 items), which had four subdimensions: critical thinking (PCT; 5 items) (e.g., “I re-read my course/study material as starting point and try to develop my own ideas about it”), rehearsal (PR; 5 items) (e.g., “I study by reading prescribed study material/my notes over and over again”), self-regulation (PS; 4 items) (e.g., “When I was confused making notes at first, I made sure I sorted it out afterwards”), and organization (PO; 4 items) (e.g., “To study, I reviewed my notes and made an outline of important concepts”). SP comprised five items adapted from Robin Lee Denalson (Robbins et al., 2004). A sample item was “Using mobile learning improved my study efficiency.” The ML construct was measured using items adapted from Venkatesh et al. (2003), comprising three dimensions. The first dimension is LMO (eight items); a sample item is “M-learning would help increase access to learning and education.” The second dimension is LSE (five items); a sample item is “I can skillfully use m-learning for my education.” The third dimension is LMT (five items); a sample item is “I am excited to use m-learning.”

### Data Analysis

After the data were collected, the data preparation process started (Malhotra, 2010), during which filled questionnaires were checked and coded. The four assumptions of SEM were fulfilled (i.e., normality, reliability, multicollinearity, and common method bias). In this study, kurtosis and skewness values were checked to verify the normality of the data; all values were within the normal range (Table 1).

**TABLE 1:** Skewness, kurtosis, and factor loading.

Serial no.	Item	Description	Skewness	Kurtosis	Factor loading
1	LMO1	Using m-learning would likely help me accomplish my studies at a time that is convenient for me.	–0.483	–0.768	0.669
2	LMO2	Using m-learning would likely help me perform my studies any place.	–0.662	–0.593	0.620
3	LMO3	Using m-learning would provide me convenience in performing my studies.	–0.599	–0.367	0.659
4	LMO4	M-learning would help increase access to learning and education.	–0.941	0.328	0.691
5	LMO5	Using mobile devices for learning enhances my personalized learning behavior.	–0.624	–0.174	0.724
6	LMO6	Using m-learning makes it possible to get real-time learning.	–0.489	–0.526	0.785
7	LMO7	Using m-learning foster more collaboration in learning.	–0.631	–0.038	0.785
8	LMO8	Using m-learning foster more social interaction in learning.	–0.581	–0.472	0.812
1	SE1	I would likely complete a learning task using a mobile device because I think I am very good at using my mobile devices.	–0.506	–0.823	0.622
2	SE2	Using m-learning I would likely feel a sense of pride.	–0.472	–0.608	0.643
3	SE3	Using m-learning I would likely feel a sense of ownership.	–0.292	–0.751	0.720
4	SE4	While using m-learning I would likely talk up the use of m-learning.	–0.533	–0.347	0.640
5	SE5	I can skillfully use m-learning for my education.	–0.880	–0.036	0.738
1	LMT1	I intend to use m-learning in my academic life.	–0.565	–0.558	0.654
2	LMT2	I am excited to use m-learning.	–0.683	–0.296	0.697
3	LMT3	I would enjoy using m-learning.	–0.511	–0.037	0.717
4	LMT4	I am interested to use m-learning frequently.	–0.608	–0.499	0.835
5	LMT5	I would enthusiastically recommend that others use m-learning.	–0.571	–0.321	0.766
1	PCT1	I often questioned things I watched (Video), heard (Audio), or read (Text) in the course to see if I found them Convincing.	–0.411	–0.572	0.624
2	PCT2	I reread my course/study material as starting point and try to develop my own ideas about it.	–0.594	–0.429	0.725
3	PCT3	Whenever I watched (Video), heard (Audio), or read (Text) an assertion or conclusion in a course, I thought about possible alternatives.	–0.561	–0.122	0.757
1	PR1	I study by reading recommended/prescribed study material/my notes over and over again.	–0.538	–0.379	0.798
2	PR2	I make list of important items for every course and memorize the list.	–0.643	–0.367	0.607
3	PR3	I memorize key words to remind me of important concepts from every course.	–0.730	–0.177	0.688
1	PS1	I ask myself questions based on my notes and other study materials to be sure I understood the material I was studying in every course.	–0.484	–0.447	0.610
2	PS2	I tried to change the way I studied in order to fit the course requirements and Instructor’s teaching style and expectations.	–0.502	–0.462	0.719
3	PS3	When studying for a course I try to determine which concepts I did not understand well.	–0.620	–0.290	0.720
4	PS4	When I was confused making notes at the first hand, I made sure I sorted it out afterward.	–0.660	–0.010	0.641
1	PO1	I make simple charts, diagrams, or tables using mobile devices to organize course material.	–0.451	–0.740	0.715
2	PO2	To study, I reviewed my notes and made an outline of important concepts.	–0.687	–0.256	0.655
3	PO3	To study, I went through my notes to find the most important ideas.	–0.590	–0.579	0.740
4	PO4	When I study the readings for a course, I outline the material to help me organize my thoughts.	–0.789	0.018	0.780
1	CLB1	Mobile learning changed my habit of studying alone.	–0.401	–0.991	0.765
2	CLB2	I actively exchange my ideas with group members/class fellows.	–0.598	–0.505	0.737
3	CLB3	I was able to develop new skills and knowledge from other members in my group/class fellows.	–0.650	–0.316	0.777
4	CLB4	I was able to develop problem solving skills through peer collaboration.	–0.521	–0.480	0.745
5	CLB5	Collaborative learning in my group is effective.	–0.672	–0.321	0.650
6	CLB6	Collaborative learning improves my academic performance.	–0.701	–0.341	0.700
1	SLB1	My learning by using/through Social Media (WhatsApp, Facebook, Twitter, YouTube…) makes learning easy.	–0.526	–0.661	0.736
2	SLB2	My learning by using/through Social Media (WhatsApp, Facebook, Twitter, YouTube…) favors problem solving/improves problem solving skills.	–0.606	–0.369	0.747
3	SLB3	My learning by using/through Social Media (WhatsApp, Facebook, Twitter, YouTube…) clarifies the learning resource.	–0.823	0.178	0.782
4	SLB4	My learning by using/through Social Media (WhatsApp, Facebook, Twitter, YouTube…) favors/makes learning sharing faster.	–0.790	0.024	0.633
5	SLB5	My learning by using/through Social Media (WhatsApp, Facebook, Twitter, YouTube…) favors/discovery of information and new knowledge useful for learning.	–0.681	–0.327	0.607
6	SLB6	Mobile learning improved my social learning ability/behavior.	–0.715	–0.377	0.649
1	SP1	Using mobile learning improved my study efficiency.	–0.752	–0.407	0.763
2	SP2	Using mobile learning enhanced my learning productivity.	–0.720	–0.090	0.831
3	SP3	By using m-learning I do my assignments and tests more skillfully.	–0.567	–0.484	0.790
4	SP4	By using m-learning my GPA improved.	–0.565	–0.533	0.607
5	SP5	By using m-learning I achieved better grades as compared to other students.	–0.656	–0.543	0.688

*ML, mobile learning; LMO, learning mobility; LMT, learning motivation; SE, self-efficacy; LB, student learning behavior; PLB, personalized learning behavior; SLB, social learning behavior; CLB, collaborative learning behavior; SP, student performance; PCT, critical thinking; PS, self-regulation; PO, organization; PR, rehearsal.*

Reliability was also ensured, as all Cronbach’s alpha values were above 0.70. Specifically, the Cronbach’s alpha values are given in Table 2.

**TABLE 2:** Reliability statistics.

Variables	Cronbach’s alpha
Mobile learning	0.796
Student learning behavior	0.912
Student performance	0.817

Furthermore, Harman’s single-factor test was used to test common method bias; this test exhibited only 29.1% of the whole variance, verifying that common method bias was not an issue. Also, the assumption of multicollinearity was met, as the variance inflation factor (VIF) values ranged from 1.3 to 2.0; thus, none exceeded the acceptable threshold value of 3.0. An SEM technique (applied using AMOS software) was utilized for a two-step data analysis approach recommended by Anderson and Gerbing (1988), involving a first-order confirmatory factor analysis (CFA) and a second-order CFA while applying SEM.

As part of the specification search, CFA was conducted with 10 first-order latent variables and 49 observed variables. A maximum likelihood estimation was used to assess the model. The first-order CFA was carried out to obtain factor loadings (FLs), squared multiple correlation (SMC) range, average variance extracted (AVE), and Cronbach’s alpha values so that any factors that might lead to poor model fit could be removed. All items were checked to determine whether their FL, AVE, or Cronbach’s alpha values were below the accepted values. The FLs of all items were within the acceptable range, so no items were removed (Table 1).

In the second-order CFA, ML, and LB were modeled as higher-order reflective constructs, while SP was modeled as a first-order construct. Furthermore, we used reliability, convergent validity, and discriminant validity as part of a measurement model analysis. The AVE values of all the first-order constructs exceeded the required threshold of 0.50, confirming the convergent validity of the first-order constructs. All FLs were significant and above the recommended thresholds, thus providing evidence of convergent validity. Three methods were used to establish discriminant validity. First, the square root of AVE was compared with the square of inter-construct correlation coefficients. The results showed that the correlation CI values of all the constructs were less than 1.00, meaning that all constructs were considerably different from one another. Thus, these results confirmed that the discriminant validity was achieved (Table 3).

**TABLE 3:** Discriminant validity.

	CR	AVE	MSV	SLB	ML	SP
SLB	0.926	0.680	0.412	**0.788**		
ML	0.887	0.723	0.557	0.737	**0.807**	
SP	0.852	0.539	0.357	0.744	0.770	**0.804**

*All diagonal bold values are square root of AVE. CR = Composite Reliability, AVE = Average Variance Extracted, MSV = Maximum Shared Variance.*

**FIGURE 2:**
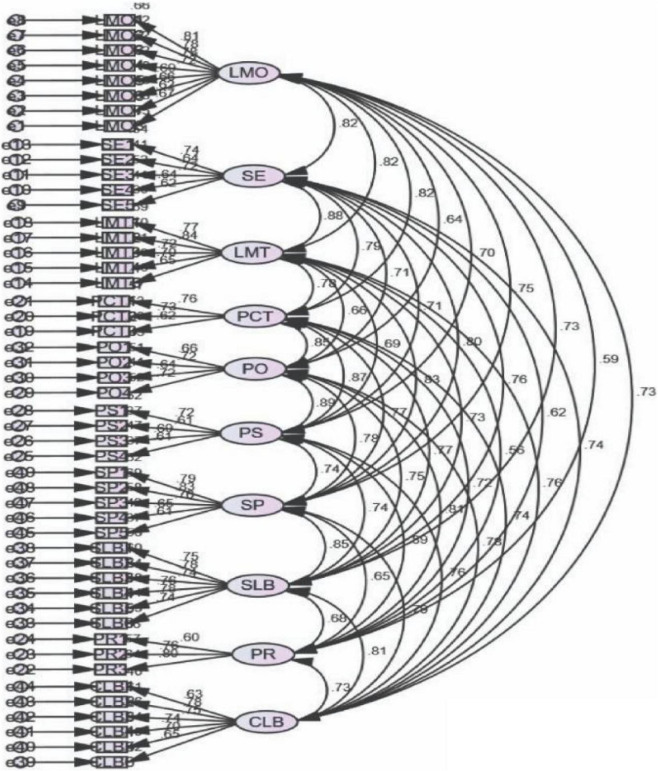
First-order-factor-analysis of all variables.

The measurement items had strong and significant FLs (*FL* ≥ 0.50), further evidencing their discriminant validity (Figure 2). The initial measurement model fit statistics were slightly below the recommended values; thus, the discriminate validity model was respecified. It is recommended to perform one modification at a time. This process is repeated until the fit indices recommended for the model are attained. Then, newly formulated modified indices were examined. The modification indices indicated the findings regarding adjustments to χ2 once high correlation was reported among error terms, which further suggests a structural path analysis. An investigation of standardized residuals is essential for adjusting a model after the compulsory removal of fundamental FL appropriateness and the standardized residual (which is problematic) > 2.58.

An assessment of the modified measurement model showed that the model fits well with the improved values of all required indices (Table 4).

**TABLE 4:** Initial measurement, final measurement, and structural models.

Fit indices	Initial measurement model	Final measurement model	Structural final specified model	Ranges and acceptance criteria	Final measurement model
CMIN/df	5.151	2.754	4.139	< 3 *Good*	Good fit
GFI	0.871	0.937	0.921	> 0.95 *Great*	Good fit
AGFI	0.817	0.902	0.937	> 0.8 *Great*	Good fit
CFI	0.907	0.971	0.947	> 0.95 *Great*	Good fit
RMSEA	0.103	0.67	0.066	0.5–0.1	Moderate fit

**FIGURE 3:**
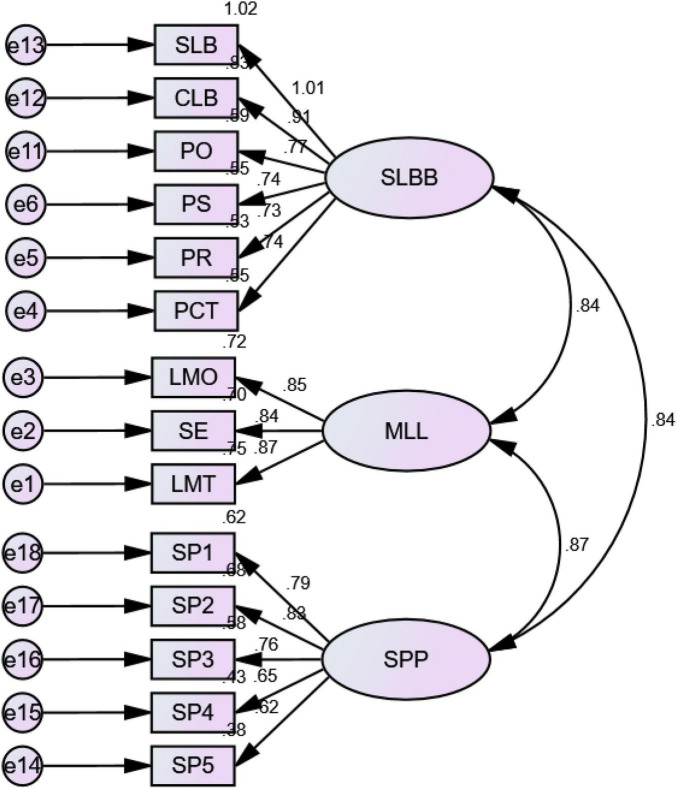
Measurement model specification.

The suggested ranges of reliability indices were reported while analyzing the measurement model: specifically, the alpha values ranged from 0.658 to 0.758 as per the internal reliability of measures. However, composite reliability (CR) ranged from 0.746 to 0.896, thus exceeding the acceptable values. The AVEs of the individual variables were evaluated to measure validity while measuring convergent validity. The values of the SMCs for all individual items were within the range of 0.766–0.785. Standardized second-order FL (Figure 3) excluded two values (i.e., SP4 0.65 and SP5 0.62). The values of all remaining items exceeded the recommended range (i.e., they were greater than 0.70). The AVE values of these items also exceeded the acceptable value (0.50), ranging from 0.538 to 0.633 for all variables. It was required that the maximum shared variance (MS) value of an item should be greater than its AVE value. The discriminant validity measurements revealed that the square roots of the AVEs of all measured variables were less than their corresponding MS values (Table 5).

**TABLE 5:** All dimensions of the study convergent and discriminant validity.

	CR	AVE	MSV	CLB	LMO	SE	LMT	PCT	PR	PS	PO	SLB	SP
CLB	0.858	0.664	0.658	**0.820**									
LMO	0.896	0.620	0.606	0.726	**0.730**								
SE	0.806	0.755	0.669	0.737	0.716	**0.804**							
LMT	0.855	0.542	0.469	0.762	0.712	0.817	**0.776**						
PCT	0.746	0.496	0.459	0.736	0.717	0.794	0.779	**0.784**					
PR	0.764	0.523	0.494	0.729	0.593	0.619	0.564	0.724	**0.813**				
PS	0.752	0.533	0.494	0.761	0.700	0.712	0.687	0.701	0.721	**0.808**			
PO	0.778	0.567	0.489	0.780	0.643	0.708	0.663	0.754	0.709	0.748	**0.784**		
SLB	0.890	0.574	0.729	0.811	0.730	0.764	0.733	0.709	0.684	0.740	0.748	**0.758**	
SP	0.851	0.537	0.729	0.788	0.752	0.798	0.719	0.708	0.648	0.735	0.775	0.754	**0.733**

*All diagonal bold values are square root of AVE. CR = Composite Reliability, AVE = Average Variance Extracted, MSV = Maximum Shared Variance.*

### Structural Model and Hypotheses Testing

After assessing the measurements of model fit, the structural model fit was evaluated to examine the theorized relationships between all endogenous and exogenous variables.

The structural model encompasses three variables comprising 11 indicators that were structured based on the theoretical/conceptual model proposed by Hayes. The model contains one exogenous variable (ML), one endogenous variable (SP), and one mediating variable (LB).

**FIGURE 4:**
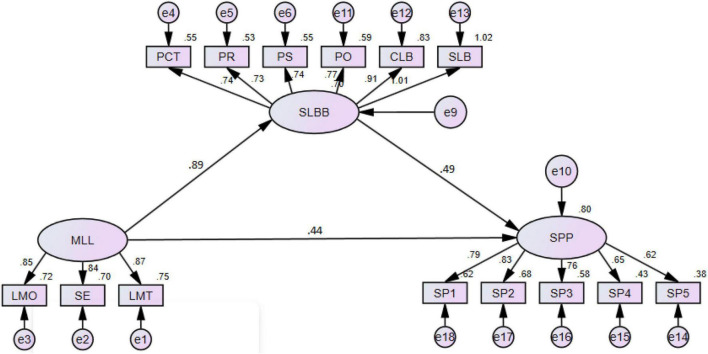
Path analysis.

The structural model had a good fit, as all indices were above the acceptable lower-limit values (Table 4). The outcomes were attained after a covariance path was added until fit indices reached the appropriate values. No paths corresponding to parameter estimates needed to be eliminated. The acceptable threshold level for the structural model is given within the model, and all four hypotheses were supported, confirming the proposed directions of significant effects. The results also showed that the overall model explained 65% (*R*2 = 0.65, *p* < 0.01) of the variance in SP (Figure 4). In section “Results,” the researcher explicates the hypothesis tests concerning the hypothesized associations between the variables of interest in this theoretical research model.

## Results

The ML has a highly significant positive relationship with SP (0.87, *p* < 0.01). Hence, H_1_ (i.e., ML is positively related to SP) is accepted. Similarly, the results showed that ML caused 91% of the change in LB. Therefore, ML had a highly significant and positive relationship with LB, thus supporting H_2_. Furthermore, the results showed that 67% of the variance in SP was due to LB. Thus, LB was significantly and positively related to SP (Table 6).

**TABLE 6:** Standardized regression weights (direct effects).

	Structural paths	Standardized regression coefficient	*P*-value	Result
H_1_	*ML* → *SP*	0.87	***	Significant (accepted)
H_2_	*ML* → *LB*	0.91	***	Significant (accepted)
H_3_	*LB* → *SP*	0.67	***	Significant (accepted)

***P < 0.01.

### Dimensional Analysis

A dimensional analysis of ML with the dimensions of SP was performed to evaluate the effects of these dimensions.

**FIGURE 5:**
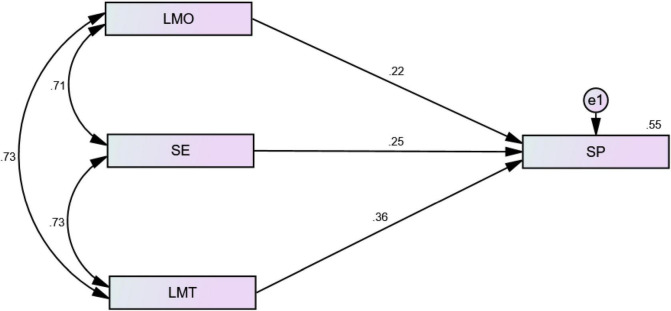
Dimensional analysis of ML with SP.

Based on the results, all hypotheses from H_1_a to H_1_c were accepted. All the individual dimensions (i.e., LMO, SE, and LMT) had significant and positive relationships with SP, as they explained 22, 25, and 36% of the variance in SP, respectively (Table 7 and Figure 5).

**TABLE 7:** Dimensional analysis of mobile learning (ML) with student performance (SP).

Variables	Estimate	*P*-value	Hypothesis
*LMO* → *SP*	0.22	***	H_1_a is accepted
*SE* → *SP*	0.25	***	H_1_b is accepted
*LMT* → *SP*	0.36	***	H_1_c is accepted

***P < 0.01.

**FIGURE 6:**
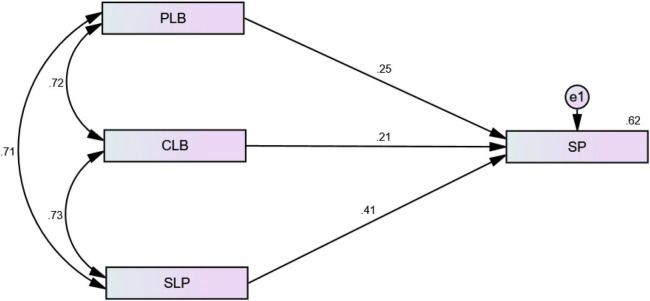
Dimensional analysis of LP with SP.

Moreover, the dimensional analysis of LB with SP was also performed, and all hypotheses from H_2_a to H_2_c were accepted based on the results. Each dimension (i.e., PLB, CLB, and SLB) had a significant positive association with SP, explaining 25, 21, and 41% of the variance in SP, respectively (Table 8 and Figure 6).

**TABLE 8:** Dimensional analysis of learning behavior (LB) with SP.

Variables	Estimate	*P*-value	Hypothesis
*PLB* → *SP*	0.25	***	H_3_a is accepted
*CLB* → *SP*	0.21	***	H_3_b is accepted
*SLB* → *SP*	0.41	***	H_3_c is accepted

***P < 0.01.

### Mediation Analyses

**FIGURE 7:**
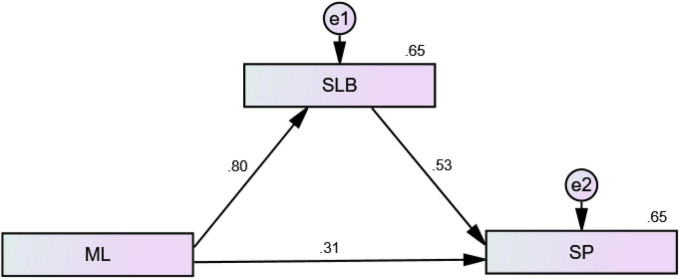
Mediation analysis.

Mediation analyses were performed with AMOS-24 by utilizing a bootstrapping technique. AMOS is designed to simultaneously estimate direct, indirect, and mediation effects (Figure 7). The significance value (which was two-tailed by bootstrapping) simultaneously showed the significance levels of the indirect, direct, and total effects. Only the standardized effect was measured for examination using AMOS. The γ value of the direct effect was equated with the γ value of the total effect to gauge the increase and decrease in the total effect.

The findings of the direct effect of *ML* → *SP* indicate that 0.74% variation in SP occurred due to ML; once LB was inserted in the path between ML and SP, the effect on SP was 31%. Based on this result, LB significantly but partially mediated the relationship between ML and SP as the association between ML and SP was reduced. Nonetheless, the relationship remained significant, and the bootstrapping two-tailed significance value was less than 0.05, which confirmed that the indirect effect in the research model was significant and that partial mediation existed. Therefore, H_4_ is accepted (Table 9).

**TABLE 9:** Standardized direct, indirect, and total effects.

H_4_	Standardized total effect		Standardized direct effect		Standardized indirect effect		Results
	Coeff	P-V	Coeff	P-V	Coeff	P-V	
*ML* → *LB* → *SP*	0.743	0.001	0.314	0.000	0.429	0.001	Partial mediation

## Discussion

The findings revealed that the learning performance of students can be enhanced by improving their LB in the present age in which novel and modern interactive, multimedia enriched, collaborative education methods are required. All hypotheses presented in this study were accepted. The SEM findings (H_1_) revealed that students who have had to learn *via* ML in the emergency situation caused by the COVID-19 pandemic obtained good outcomes, sometimes improving on their pre-COVID performance. Therefore, it can be said that the ML-based solution to the stoppage of face-to-face education has been viable. Based on the benefits that have been attained from ML in enhancing SP (either directly or indirectly), universities should strive to provide ML so that students can further develop their ML behaviors. The efforts of universities in this direction seem to have been fruitful and should be continued in the form of blended learning when COVID-19 lockdown ends. The findings presented in this study support the notion that implementing ML at higher education institutions allows students to improve their LB from anywhere at any time with anyone, thus validating the theory of learning in the mobile age (Sharples et al., 2016).

Furthermore, we attempted to fill the gap identified by Li et al. (2019) by performing the dimensional analyses of the relationships between ML and LB and between LB and SP. The findings revealed that H_1_a conforms to the findings of Sharples et al. (2016), which showed that LMO increases the outcomes of students. This is mainly because students who are mobile and can freely learn whenever it is convenient for them (and learn with any device and in any way with tutors, peers, or large social communities) exhibit improvements in their academic performance. The results substantiate the theory of learning in the mobile age, which emphasizes the role of the mobility of learners in improving student outcomes.

The findings related to H_1_b conform to the findings of Tsai (2012), indicating that LSE enhances SP. This finding also supports the findings of Robbins et al. (2004), Liang and Tsai (2008), and Tsai (2012), as it shows that LSE enhances SP. The findings of this study also corroborate the findings of Chemers et al. (2001), who showed that LSE enhances SP. These results support the Unified Theory of Acceptance and the usage of the technology acceptance model of the technology, which is based on the theory of reasoned action (Ajzen and Fishbein, 1975).

Similarly, the findings related to H_1_c revealed that motivated students perform better than other students in ML. This suggests that frequent ML users perform better than other students. Moreover, students who skillfully and productively engage in ML are likely to enthusiastically recommend it to others so they can also improve their academic performance. These findings align with those of other researchers (Malone and Lepper, 1987; Li et al., 2018b) who showed that strong motivation was required to obtain outstanding academic performance. The findings are also in line with the claim of the self-regulated learning theory that learning strategy and motivation directly impact learning outcomes.

The findings related to H_2_ specified that ML enhances LB by fostering organized studying in small groups and large communities. This fact might make learners more engaged, thus improving their results. This finding conforms to the findings of Shorfuzzaman and Alhussein (2016) that mobile devices improve the LB of students by encouraging them to use their time efficiently. The findings related to H_3_ revealed that LB enhances SP, specifically, after improving their LB, students tend to exercise self-regulation when studying with their peers and social groups; they also obtain more academic achievements due to their personalized, social, and collaborative efforts.

Furthermore, the findings of the dimensional analysis for H_3_a indicated that PLB enhances SP, thus filling the gap identified by Li et al. (2019). Students who are good critical thinkers, rehearse their lessons, are more self-regulated in their studies, and can schedule their studies effectively show better academic performance than other students due to their more effective LB. In other words, they know what to study, when to study, and how to study within the ML context. These findings align with the theory of planned behavior, which emphasizes the fact that self-regulated, organizational behavior improves the outcomes of students.

The findings for H_3_b indicated that CLB enhanced SP, which supports previous findings (Al-Rahmi et al., 2015). Engaging in peer-to-peer learning and exchanging ideas in group settings foster new skills, knowledge, and problem-solving skills, thereby enhancing academic performance through CLB. These findings are in accordance with the collaborative learning theory as described previously (Sarrab et al., 2013). This theory states that CLB improves learning outcomes by accelerating interactions, facilitating participation among learners in groups, improving peer-to-peer communication, promoting knowledge-sharing, and improving problem-solving in groups within an ML context.

The findings of this study verify the claim of social constructivists that ML theory is a vigorous process of acquiring skills and enhancing knowledge through group learning. The findings for H_3_c support the social learning theory, which links behaviorist learning theories and cognitive learning theories as it encompasses memory, motivation, and attention. This theory claims that learners do not learn in isolation; instead, they learn from the behavioral, cognitive, and environmental effects of their interactions with others.

The findings also verify the social constructivist approach to ML, according to which learning is the process of acquiring skills and enhancing knowledge within a community. The results also indicate that students were able to improve their grades if they felt that ML enhanced their problem-solving skills, comprehension of learning resources, and discovery of knowledge by learning in online communities through social media. These findings support the results reported by Kreijns et al. (2003).

Finally, the findings for H_4_ revealed that ML is a strong predictor of SP. This relationship can be intensified by adding LB as a mediating factor because it increases critical thinking, self-regulation, organization, and peer-to-peer and community learning.

## Conclusion and Implications

This study applied a new perspective for predicting SP and makes three major contributions to the literature. First of all, it complements the ML literature and provides new insights by evaluating the individual impacts of LMT, self-efficacy, and mobility, as well as their combined effect as a composite ML variable. New realities arose, such as the finding that the internal motivation of learners can be harnessed to improve LB and, in turn, the academic performance of students. Second, the results revealed that the freedom to learn from anywhere at any time with anyone enables students with personal soft skills to improve their LB by fostering their growth as independent learners and thinkers. Third, this study does not only investigate the mediated impact of LB but also thoroughly studies the dimensional effect of MLB on SP as well as LB on SP. In this way, this study contributes to behavioral learning theories by integrating the theory of collaborative learning, sociocultural theory, and the theory of learning in the mobile age; uncovering the value of ML; and revealing the necessity of ML environments and facilitation by institutions to improve SP. These findings can be utilized for face-to-face and distance learning education by virtual universities that are anxious to educate the millennial generation, which is accustomed to spending time on mobile devices. These findings also highlight the importance of creative teachers who can adapt to changing mobile technologies to improve their skills.

Furthermore, policymakers should consider that ML motivates digital natives and implement policies for installing Information and Communication Technology (ICT) and mobile technologies according to the psychology of students. This information highlights that higher education institutions can best fulfill their vital roles when they offer a better ICT and ML setting that empowers educators by helping them become creative and innovative learners who will lead the students to success within the university and beyond.

The COVID-19 pandemic has forced educational institutions to shift from offline to online instruction by adopting modern technology. Online teaching and learning were considered the panacea for the crisis caused by school closures after the outbreak of COVID-19. The new circumstances appeared to increase the importance of ML, which has now become an inevitable part of the lives of students. Moreover, this catastrophe has shown us several previously unknown benefits of online teaching and learning. It seems, however, that the traditional methods of teaching and learning have become less effective as new technologies continue to emerge and develop (Zare Bidaki et al., 2013). With the help of online teaching modes, teachers can instruct many students from any part of the world at any time.

The inevitability of ML has shown that even in the coming post-COVID period, e-learning should be used alongside traditional methods to foster academic efficiency and effectiveness. In this mobile age, all educational institutions need to consider using different online teaching methods and determining how these methods can be used most effectively. However, the findings of this research pose certain challenges that may hinder ML adoption and that educational institutions need to address through policy revisions. These challenges may be associated with the lack of digital skills of both teachers and learners, the lack of interactivity and motivation of learners, the lack of social and cognitive presence of teachers, the lack of human interaction between teachers and students, and the lack of communication among the students. One more important challenge may be related to current academic evaluation methods, as learning is spreading across multiple new settings. Therefore, the methods used to define and evaluate effective learning need to be reconsidered.

This study has some limitations. For one, this study was cross-sectional, and future studies should employ longitudinal or time lag designs to better investigate causal relationships. Moreover, the study population comprised university students only. Other categories of students (e.g., primary school students and college students) should be included in future research. Moreover, different learning outcomes of ML, such as student satisfaction and student engagement, should be investigated. Also, specific dispositional factors such as student/teacher personality, availability of financial resources, working status, and gender should be used as moderators by future researchers to more thoroughly explain this mode of learning. Future research should also be conducted on students in remote or low living areas in developing and underdeveloped countries because these students face many physical and non-physical constraints, including the unavailability of personal mobile devices, electricity shutdowns, the unavailability of the Internet, and poverty.

## Data Availability Statement

The original contributions presented in the study are included in the article/supplementary material, further inquiries can be directed to the corresponding author.

## Ethics Statement

The studies involving human participants were reviewed and approved by the Riphah International University. The patients/participants provided their written informed consent to participate in this study.

## Author Contributions

ZW and AQ contributed in conceptualization of literature review and data analysis. AA and MA contributed in data collection and formal analysis. AQ and XL contributed supervising and validation of the original draft. All authors contributed to the article and approved the submitted version.

## Conflict of Interest

The authors declare that the research was conducted in the absence of any commercial or financial relationships that could be construed as a potential conflict of interest.

## Publisher’s Note

All claims expressed in this article are solely those of the authors and do not necessarily represent those of their affiliated organizations, or those of the publisher, the editors and the reviewers. Any product that may be evaluated in this article, or claim that may be made by its manufacturer, is not guaranteed or endorsed by the publisher.

## References

[B1] Admiraal W. Kester L. Janssen C. de Jonge M. Louws M. Post L. (2018). “Personalizing learning with mobile technology in secondary education,” in *Proceedings of the International Association for Development of the Information Society*, (Lisbon).

[B2] Ajzen I. Fishbein M. (1975). A Bayesian analysis of attribution processes. *Psychol. Bull.* 82, 261–277. 10.1037/h0076477

[B3] Alghazi S. S. Kamsin A. Almaiah M. A. Wong S. Y. Shuib L. (2021). For Sustainable application of mobile learning: an extended UTAUT model to examine the effect of technical factors on the usage of mobile devices as a learning tool. *Sustainability* 13:1856. 10.3390/su13041856

[B4] Alqahtani M. Mohammad H. (2015). Mobile applications’ impact on student performance and satisfaction. *TOJET Turkish Online J. Educ. Technol.* 14 102–112.

[B5] Al-Rahmi W. M. Othman M. S. Yusuf L. M. (2015). Exploring the factors that affect student satisfaction through using e-learning in Malaysian higher education institutions. *Mediterr. J. Soc. Sci.* 6:299.

[B6] Anderson J. C. Gerbing D. W. (1988). Structural equation modeling in practice: a review and recommended two-step approach. *Psychol. Bull.* 103:411.

[B7] Bandura A. (1997). *Theoretical Perspectives. Self–Efficacy: The Exercise of Control.* 1–35.

[B8] Baytiyeh H. (2018). Online learning during post-earthquake school closures. *Disaster Prev. Manag.* 27 215–227. 10.1108/dpm-07-2017-0173

[B9] Behera S. K. (2013). E- and M-learning: a comparative study. *Int. J. New Trends Educ. Their Implic.* 4, 65–78.

[B10] Bidin S. Ziden A. A. (2013). Adoption and application of mobile learning in the education industry. *Procedia Soc. Behav. Sci.* 90 720–729.

[B11] Caprara G. V. Barbaranelli C. Pastorelli C. Bandura A. Zimbardo P. G. (2000). Prosocial foundations of children’s academic achievement. *Psychol. Sci.* 11 302–306. 10.1111/1467-9280.00260 11273389

[B12] Chemers M. M. Hu L. T. Garcia B. F. (2001). Academic self-efficacy and first year college student performance and adjustment. *J. Educ. Psychol.* 93, 55–64. 10.1037/0022-0663.93.1.55

[B13] Cruz-Flores R. López-Morteo G. (2008). “A model for collaborative learning objects based on mobile devices,” in *Proceedings of the 2008 Mexican International Conference on Computer Science.* (Mexicali), 89–95.

[B14] Czerniewicz L. (2020). *What we Learnt From “Going Online” During University Shutdowns in South Africa. Philon Edtech.* 1–10. *.

[B15] del Barco B. L. Mendo-Lázaro S. Felipe-Castaño E. del Río M. I. P. Fajardo-Bullón F. (2017). Team potency and cooperative learning in the university setting. *Rev. Psicodidáctica (English Ed.)* 22 9–15. 10.3389/fpsyg.2018.00326 29593622 PMC5859103

[B16] Dillon P. Wang R. Tearle P. (2007). Cultural disconnection in virtual education. *Pedagogy Cult. Soc.* 15 153–174. 10.1080/14681360701403565

[B17] Elfeky A. I. M. Masadeh T. S. Y. (2016). The effect of mobile learning on Students’ achievement and conversational skills. *Int. J. High. Educ.* 5 20–31.

[B18] Eyles A. Gibbons S. Montebruno P. (2020). *Covid-19 School Shutdowns: What Will They do to Our Children’s Education? Economic and Social Research Council.* London: London School of Economics and Political Science, 1–8.

[B19] Hao S. Dennen V. P. Mei L. (2017). Influential factors for mobile learning acceptance among Chinese users. *Educ. Technol. Res. Dev.* 65 101–123. 10.1007/s11423-016-9465-2

[B20] Hashim A. S. Ahmad W. F. Rohiza A. (2011). “Mobile learning course content application as a revision tool: the effectiveness and usability,” in *In Proceedings of the 2011 International Conference on Pattern Analysis and Intelligence Robotics*, Vol. 2 (Kuala Lumpur), 184–187.

[B21] Hatcher L. (1994). *A Step-by-Step Approach to Using the SAS System for Factor Analysis and Structural Equation Modeling.* New York City, NY: SAS Institute, Inc.

[B22] Ismail I. Azizan S. N. Gunasegaran T. (2016). Mobile learning in malaysian universities: are students ready? *Int. J. Interact. Mobile Technol.* 10 17–23.

[B23] Jaradat R. M. (2014). Students’ attitudes and perceptions towards using m-learning for French language learning: a case study on Princess Nora University. *Int. J. Learn. Manag. Syst.* 2 33–44. 10.12785/ijlms/020103

[B24] Kabir F. S. Kadage A. T. (2017). ICTs and educational development: the utilization of mobile phones in distance education in Nigeria. *Turkish Online J. Distance Educ.* 18 63–76. 10.17718/tojde.285716

[B25] Keskin N. O. Metcalf D. (2011). The current perspectives, theories and practices of mobile learning. *Turkish Online J. Educ. Technol.* 10 202–208. 10.3390/ijerph16214206 31671592 PMC6862580

[B26] Khaddage F. Lanham E. Zhou W. (2009). A mobile learning model for universities. *Int. J. Interact. Mobile Technol.* 3 18–23. 10.3991/ijim.v3s1.949

[B27] Kilis S. (2013). Impacts of mobile learning in motivation, engagement and achievement of learners: review of literature. *Gaziantep Univ. J. Soc. Sci.* 12 375–383.

[B28] Koole M. (2014). Using smart mobile devices in social-network-based health education practice: a learning behavior analysis. *Nurse Educ. Today* 34 958–963. 10.1016/j.nedt.2014.01.013 24568697

[B29] Kreijns K. Kirschner P. A. Jochems W. (2003). Identifying the pitfalls for social interaction in computer-supported collaborative learning environments: a review of the research. *Comput. Hum. Behav.* 19 335–353. 10.1016/s0747-5632(02)00057-2

[B30] Kukulska-Hulme A. Traxler J. (2007). “Designing for mobile and wireless learning,” in *Rethinking Pedagogy for a Digital Age: Designing and Delivering E-Learning*, eds Beetham H. Sharpe R. (London: Routledge), 180–192.

[B31] Li K. Lee L.Y.-K. Wong S.-L. Yau I.S.-Y. Won B.T.-M. (2019). The effects of mobile learning for nursing students: learning motivation, social interaction and study performance. *Int. J. Mobile Learn. Organ.* 13 51–67.

[B32] Li K. C. Lee L. Y. Wong S. L. Yau I. S. Wong B. T. (2018a). Preference and Readiness of Nursing Students for Mobile Learning. *Innov. Open Flex. Educ.* 1 97–107. 10.1007/978-981-10-7995-5_9

[B33] Li K. C. Lee L. Y.-K. Wong S.-L. Yau I. S.-Y. Wong B. T.-M. (2018b). Effects of mobile apps for nursing students: learning motivation, social interaction and study performance. *Open Learn.* 33 99–114.

[B34] Liang J. C. Tsai C. C. (2008). Internet self-efficacy and preferences toward constructivist internet-based learning environments: a study of pre-school teachers in Taiwan. *J. Educ. Technol. Soc.* 11, 226–237.

[B35] Liguori E. Winkler C. (2020). From offline to online: challenges and opportunities for entrepreneurship education following the COVID-19 pandemic. *SAGE J.* 3 1–6.

[B36] MacCallum K. Jeffrey L. (2009). Identifying discriminating variables that determine mobile learning adoption by educators: an initial study, Same places, different spaces. *Proc. Ascilite Auckland* 1 602–608.

[B37] Malhotra N. K. (2010). *Marketing Research: An Applied Orientation*, 7th Edn. London: Pearson.

[B38] Malone T. Lepper M. R. (1987). “Making learning fun: a taxonomic model of intrinsic motivations for learning,” in *Aptitude, Learning and Instruction III:Conative and Affective Process Analyses*, eds Snow R. E. Farr M. J. (Hilsdale, NJ: Erlbaum), 223–253.

[B39] Means B. Toyama Y. Murphy R. Bakia M. Jones K. (2000). *Evaluation of Evidence–Based Practices in Online Learning: A Meta-Analysis and Review of Online Learning Studies. Project Report. Centre for Learning Technology.* Washington, DC: United States Department of Education, 1–55.

[B40] Naciri A. Baba M. A. Achbani A. Kharbach A. (2020). Mobile learning in Higher education: unavoidable alternative during COVID-19. *Aquademia* 4 1–2. 10.1504/ijmc.2023.10042533

[B41] Nilsson B. (2016). *The State of Personalized Learning in The Real World of Education: Survey and Infographic.* San Jose, CA: Extreme.

[B42] Pajares F. (2002). Gender and perceived self-efficacy in self-regulated learning. *Theory Pract.* 41 116–125. 10.1207/s15430421tip4102_8 33486653

[B43] Pellas N. Peroutseas E. Kazanidis I. (2013). “Virtual communities of inquiry (VCoI) for learning basic algorithmic structures with Open Simulator and Scratch4OS: a case study from the Secondary education in Greece,” in *Proceedings of the 6th Balkan Conference in Informatics*, (New York, NY), 187–194.

[B44] Qureshi E. Morton L. Antosz E. (2002). An interesting profile-university students who take distance education courses show weaker motivation than on-campus students. *Online J. Distance Learn. Adm.* 5 1–7.

[B45] Razzaq A. Samiha Y. T. Anshari M. (2018). Smartphone habits and behaviors in supporting students self-efficacy. *Int. J. Emerg. Technol. Learn.* 12 94–109. 10.3991/ijet.v13i02.7685

[B46] Reed M. S. Evely A. C. Cundill G. Fazey I. Glass J. Laing A. (2010). What is social learning? *Ecol. Soc.* 15 1–10.

[B47] Robbins S. B. Lauver K. Le H. Davis D. Langley R. Carlstrom A. (2004). Do psychosocial and study skill factors predict college outcomes? A meta-analysis. *Psychol. Bull.* 130 261–288. 10.1037/0033-2909.130.2.261 14979772

[B48] Sarrab M. Al-Shihi H. Rehman O. M. (2013). Exploring major challenges and benefits of m-learning adoption. *Curr. J. Appl. Sci. Technol.* 3 826–839. 10.9734/bjast/2013/3766

[B49] Schwen T. M. Hara N. (2003). Community of practice: a metaphor for online design? *Inform. Soc.* 19 257–270. 10.1080/01972240309462

[B50] Sharples M. Taylor J. Vavoula G. (2016). “A theory of learning for the mobile age,” in *SAGE Handbook of E-Learning Research*, 2nd Edn, eds Haythornthwaite C. Andrews R. Fransman J. Andrews R. N. L. Meyers E. M. (Thousand Oaks, CA: SAGE), 63–81. 10.4135/9781473955011.n4

[B51] Shorfuzzaman M. Alhussein M. (2016). Modeling learners’ readiness to adopt mobile learning: a perspective from a GCC higher education institution. *Mobile Inform. Syst.* 2016 1–10. 10.1155/2016/6982824

[B52] Srinivas H. (2011). *What is Collaborative Learning?.* Kobe: The Global Development Research Center.

[B53] Thomas M. Cathy R. (2020). Education, the science of learning, and the COVID-19 crisis. *Prospects* 1 87–90. 10.1007/s11125-020-09468-z PMC7246955 32836416

[B54] Tinto V. (1994). *Building Learning Communities for New College Students: A Summary of Research Finding of the Collaborative Learning Project.* Washington, DC: PSU: National Center on Postsecondary Teaching, Learning, and Assessment, 1–8.

[B55] Toquero C. M. (2020). Challenges and Opportunities for Higher Education Amid the COVID-19 Pandemic: the Philippine Context. *Pedagog. Res.* 5 1–5.

[B56] Traxler M. J. (2005). Plausibility and verb subcategorization in temporarily ambiguous sentences: evidence from self-paced reading. *J. Psycholinguistic Res.* 34 1–30. 10.1007/s10936-005-3629-2 15968918

[B57] Tsai C.-C. (2012). The development of epistemic relativism versus social relativism via online peer assessment, and their relations with epistemological beliefs and internet self-efficacy. *J. Educ. Technol. Soc.* 15 309–316.

[B58] Usak M. Masalimova A. R. Cherdymova E. I. Shaidullina A. R. (2020). New playmaker in science education: COVID-19. *J. Baltic Sci. Educ.* 19 180–185. 10.33225/jbse/20.19.180

[B59] Valk J.-H. Rashid A. T. Elder L. (2010). Using mobile phones to improve educational outcomes: an analysis of evidence from Asia. *Int. Rev. Res. Open Distrib. Learn.* 11 117–140. 10.19173/irrodl.v11i1.794

[B60] Venkatesh V. Morris M. G. Davis G. B. Davis F. D. (2003). User acceptance of information technology: toward a unified view. *MIS Q.* 27 425–478. 10.2307/30036540

[B61] Vygotsky L. S. (1978). Socio-cultural theory. *Mind Soc.* 6 52–58.

[B62] Wang M. Shen R. Novak D. Pan X. (2009). The impact of mobile learning on Students’ learning behaviours and performance: report from a large blended classroom. *Br. J. Educ. Technol.* 40 673–695. 10.1111/j.1467-8535.2008.00846.x

[B63] Wang S.-L. Wu P.-Y. (2008). The role of feedback and self-efficacy on web-based learning: the social cognitive perspective. *Comput. Educ.* 51 1589–1598. 10.1016/j.compedu.2008.03.004

[B64] Welsh M. Parke R. D. Widaman K. O’Neil R. (2001). Linkages between children’s social and academic competence: a longitudinal analysis. *J. Sch. Psychol.* 39 463–482. 10.1016/s0022-4405(01)00084-x

[B65] Wentzel K. R. (1991a). Relations between social competence and academic achievement in early adolescence. *Child Dev.* 62, 1066–1078. 10.1111/j.1467-8624.1991.tb01589.x 1756656

[B66] Wentzel K. R. (1991b). Social competence at school: relations between social responsibility and academic achievement. *Rev. Educ. Res.* 61 1–24.

[B67] Wentzel K. R. (1993). Does being good make the grade? Social behaviour and academic competence in middle school. *J. Educ. Psychol.* 85 357–364. 10.1186/s12889-021-10355-1 33602184 PMC7893771

[B68] Westera W. (2011). On the changing nature of learning context: anticipating the virtual extensions of the world. *J. Educ. Technol. Soc.* 14 201–212.

[B69] Yang X. Li J. Guo X. Li X. (2015). Group interactive network and behavioral patterns in online English-to-Chinese cooperative translation activity. *Internet High. Educ.* 25 28–36. 10.1016/j.iheduc.2014.12.003

[B70] Yukseloglua S. M. Karaguven H. (2013). Academic motivation levels of technical high school students. *Proc. Soc. Behav. Sci.* 106 282–288. 10.1016/j.sbspro.2013.12.033

[B71] Zare Bidaki M. Naderi F. Ayati M. (2013). Effects of mobile learning on paramedical students’ academic achievement and self-regulation. *Future Med. Educ. J.* 3 24–28.

[B72] Zins J. E. Weissberg R. P. Wang M. C. Walberg H.J. (eds.) (2004). *Building Academic Success Through Social and Emotional Learning: What Does the Research Say?.* New York, NY: Teachers College Press.

